# Ruptured renal arteriovenous malformation successfully treated by catheter embolization: a case report

**DOI:** 10.1186/1756-0500-7-19

**Published:** 2014-01-09

**Authors:** Nobuhiro Takeuchi, Yusuke Nomura

**Affiliations:** 1 Department of Internal Medicine, Kawasaki Hospital, Kobe, Japan

**Keywords:** Renal arteriovenous malformation, Catheter embolization

## Abstract

**Background:**

Renal arteriovenous fistula (RAVF) is a comparatively rare malformation. Here, we report a case of ruptured RAVF that was successfully treated by catheter embolization.

**Case presentation:**

An 89-year-old female was transferred to our institution with massive gross hematuria in March 2011. Plain abdominal computed tomography (CT) revealed dilated left renal pelvis with high-density contents. Hematoma was suspected. Subsequent plain abdominal magnetic resonance imaging revealed left hydronephrosis and blood retention in the dilated left renal pelvis. No renal or ureteral cancer was evident. Hematuria was conservatively treated using hemostatic agents but hematuria persisted. Repeated urinary cytology revealed no malignant cells. On day 9, the patient went into septic and/or hemorrhagic shock. Fluid and catecholamine infusion, blood transfusion, and antibacterial drugs were rapidly initiated, and the patient’s general condition gradually improved. Contrast-enhanced abdominal CT revealed marked expansion of the hematoma in the renal pelvis and microaneurysms in the segmental arteries of the left kidney. Inflammation improved, and a left double-J stent was inserted. Selective renal angiography revealed RAVF with microaneurysms in the left segmental arteries; therefore, catheter embolization using metallic coils was performed, which resolved hematuria.

**Conclusion:**

We report a case of ruptured renal arteriovenous malformation, which was successfully treated by catheter embolization.

## Background

Renal arteriovenous fistula (RAVF) is a comparatively rare malformation that accounts for < 1% of arteriovenous fistulas among the general population [1]. RAVF may either be congenital or acquired [1]. Acquired RAVF occurs because of trauma, biopsy, surgery, infection, or malignant tumors. We report a case of ruptured renal arteriovenous malformation, which was successfully treated by catheter embolization.

## Case presentation

**Figure 1:**
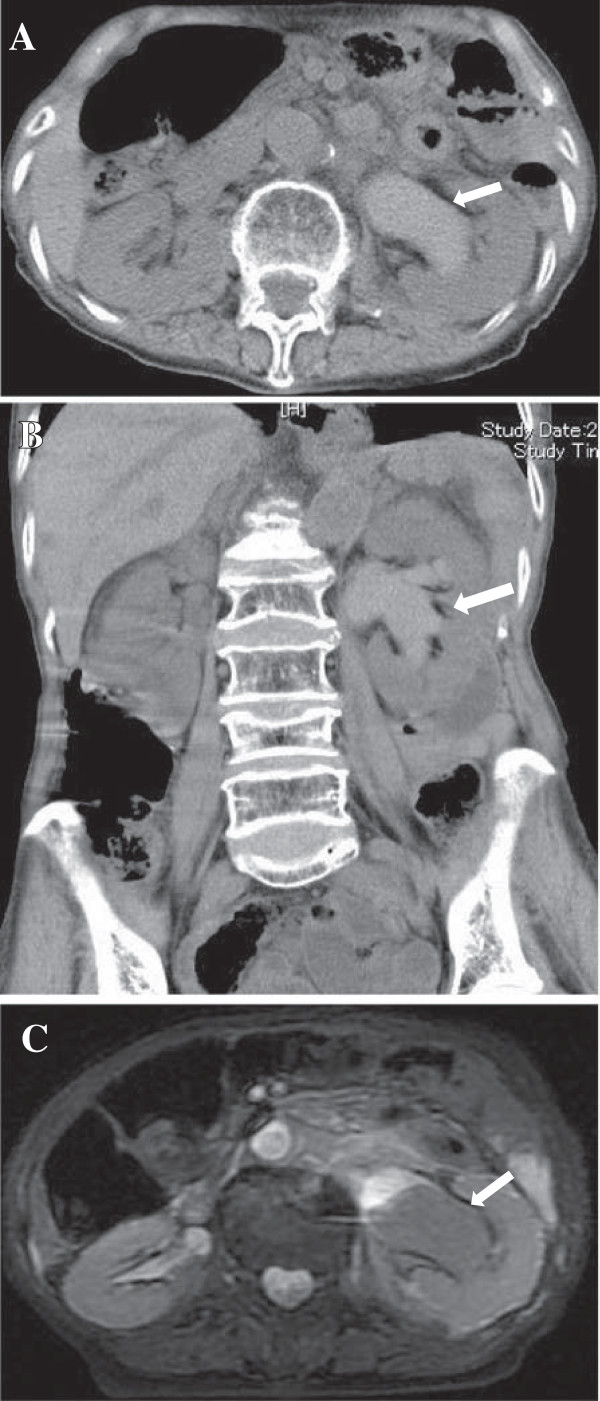
**Plain abdominal computed tomography and magnetic resource imaging.** Plain abdominal computed tomography revealed dilated left renal pelvis with retention of high-density contents (**A**: axial view, **B**: coronal view). T2-weighted abdominal magnetic resonance imaging revealed dilated left renal pelvis with retention of low-intensity fluid, which suggested the presence of blood **(C)**.

An 89-year-old female was transferred to our institution with massive gross hematuria in March 2011. The patient’s medical history included uterine cervical cancer for which surgical treatment was performed at the age of 45 and gastric cancer treated by total gastrectomy at the age of 55. Medical history did not include renal biopsy, renal injury, percutaneous nephrolithotomy, or no trauma at the flank region. On admission, blood pressure was 143/67 mmHg, heart rate was 83 beats/min, body temperature was 37.2°C, and oxygen saturation was 99% on room air. On clinical examination, weight was 45 kg, height was 143 cm, and body mass index was 22.0 kg/m^2^. Inspection of the palpebral conjunctiva revealed evidence of anemia. Blood chemical analyses were shown in Table 1. A urinalysis revealed urine protein level of 3+, urine occult blood level of 3+, white blood cell count of 10–19/HPF, and red blood cell count of >100/HPF. Plain abdominal computed tomography (CT) revealed dilated left renal pelvis with high-density contents and hematoma was suspected (Figure 1A, B). Contrast enhanced CT was not performed because this condition was not considered to have association with renal vascular diseases. Subsequent plain abdominal magnetic resonance imaging revealed left hydronephrosis and blood retention in the dilated left renal pelvis. No cancer in the renal pelvis or ureter was detected (Figure 1C).

**Table 1:** Laboratory data on admission

**Hematology**	
WBC	13,700/μl
RBC	338 × 10^4^/μl
Hb	9.7 g/dl
Ht	28.7%
MCV	85.0 fl
PTL	21.9 × 10^4^/μl
**Biochemistry**	
TP	6.0 g/dl
Alb	3.5 g/dl
T-Bil	1.4 mg/dl
*γ*GTP	91U/l
ALP	632 IU/l
AST	22 IU/l
ΛLT	17 IU/l
LDH	234 IU/l
BUN	26.9 mg/dl
Cr	0.87 mg/dl
CK	48 IU/l
Na	141 mEq/1
K	4.0 mEq/l
Cl	105 mEq/l
Glu	172 mg/dl
CRP	3.2 mg/dl
**Coagulation**	
APTT	31.6 sec
PT	110%
FDP	18.8 μg/ml
D-dimer	11.5 μg/ml

**Figure 2:**
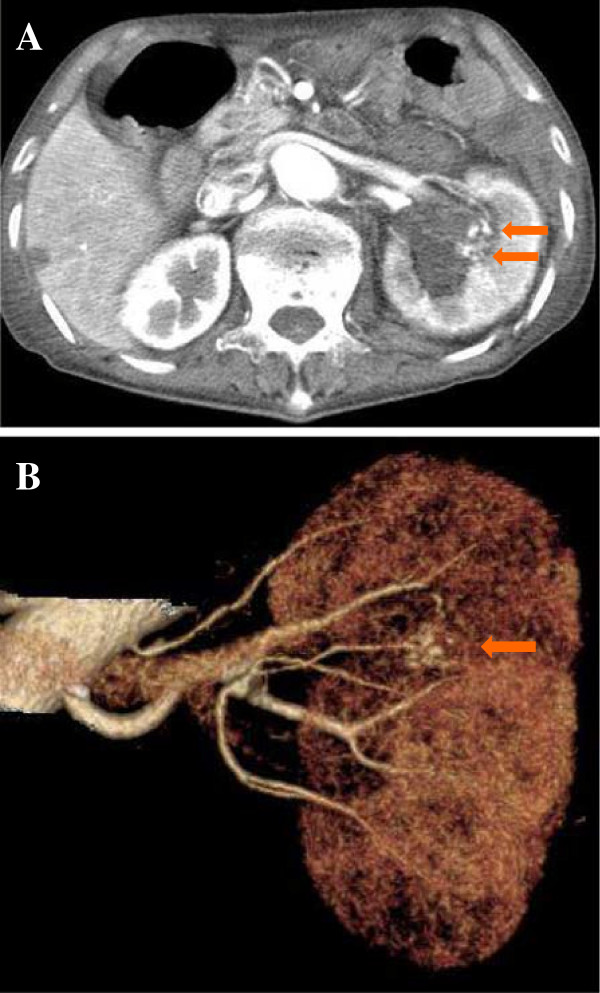
**Contrast-enhanced abdominal computed tomography.** Contrast-enhanced abdominal computed tomography revealed expansion of hematoma in the pelvis and small aneurysms (arrow) in the segmental arteries of the left kidney (**A**: axial view, **B**: threedimensional construction).

Despite conservative treatment with hemostatic agents (carbazochrome sodium sulphate and tranexamic acid), hematuria persisted. Repeated urinary cytology revealed no malignant cells. On day 9 after admission, consciousness deteriorated, systolic blood pressure decreased to 60 mmHg, and a high-grade fever of 39.0°C developed. Laboratory blood tests revealed a markedly elevated white blood cell count (35,000/μL), markedly elevated C-reactive protein levels (32.5 mg/dL), and moderate anemia (red blood cell count, 206 × 10^4^/μL; hemoglobin, 6.0 g/dL). The patient went into septic and/or hemorrhagic shock. Therefore, fluid and catecholamine infusion, blood transfusion, and antibacterial drugs were rapidly initiated. Consciousness level and circulatory condition gradually improved. Repeated blood culture prior to administration of antibacterial drugs revealed no evidence of bacterial infection. On day 10 after admission, abdominal ultrasonography revealed expansion of hematoma in the left renal pelvis. Contrast-enhanced abdominal CT confirmed marked expansion of hematoma in the left renal pelvis and identified microaneurysms in the segmental arteries of the left kidney (Figure 2A, B).

**Figure 3:**
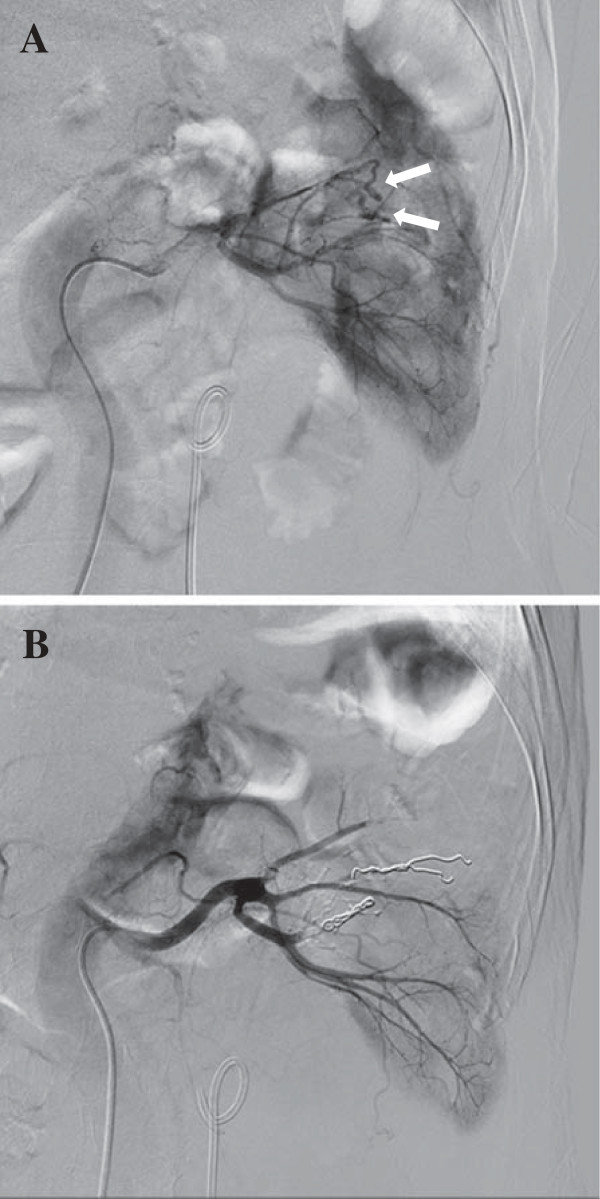
**Selective renal angiography.** Selective renal angiography revealed renal arteriovenous fistula with microaneurysms (arrows) in the left segmental arteries **(A)**. Catheter embolization using metallic coils was successfully performed **(B)**.

**Figure 4:**
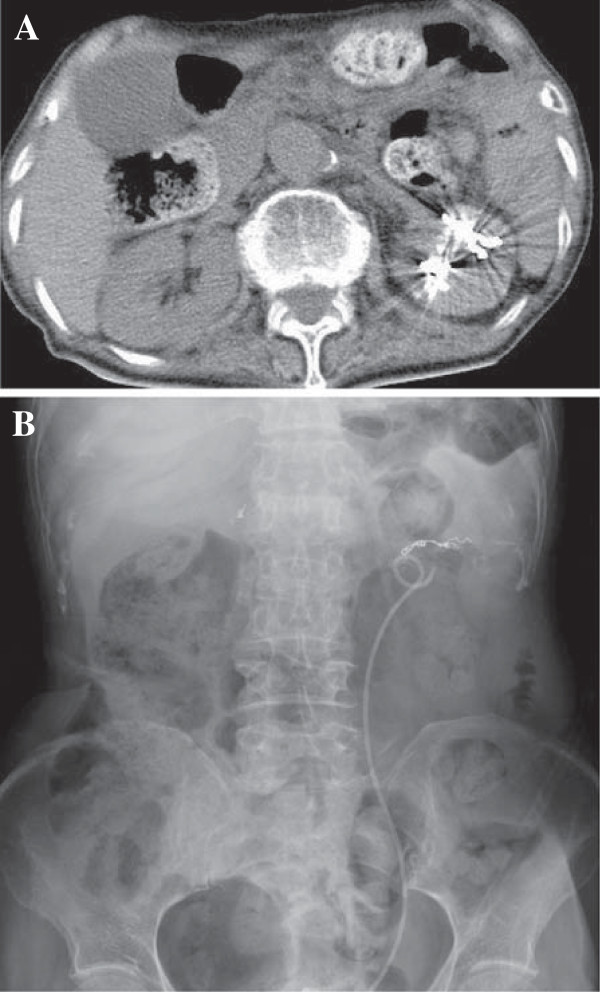
**Plain abdominal computed tomography revealed resolution of hematoma in the left renal pelvis and presence of metallic coils (A).** Abdominal radiography revealed a well-positioned double-J stent **(B)**.

Inflammation improved and a left double-J stent was inserted to improve hydronephrosis. Selective renal angiography that was simultaneously performed revealed renal arteriovenous malformation with microaneurysms in the left segmental arteries (Figure 3A). Catheter embolization using metallic coils was then successfully performed (Figure 3); thus, hematuria ceased. Subsequent plain abdominal CT confirmed the absence of hematoma in the left renal pelvis (Figure 4A). Abdominal radiography revealed a well-positioned double-J stent (Figure 4B). The subsequent clinical course was uneventful. No postembolization syndrome (fever, lumbago, elevation of lactase dehydrogenase, or hematuria) was observed. The patient was ambulatory on discharge.

## Discussion

On angiographic examination, RAVF may be classified as cirsoid or aneurysmal [2]. Cirsoid-type RAVF consists of multiple small and dilated arteriovenous communications with multiple feeding arteries and draining veins. Aneurysmal-type RAVF consists of a single feeding artery and a single draining vein. RAVF may either be congenital or acquired [1]. Congenital RAVF primarily consists of the cirsoid type and is more often observed in females than males (male to female ratio: 1:2) aged between 20 and 40 years [3]. The right kidney is the most frequent site of congenital RAVF development [4]. In contrast, acquired RAVF is primarily aneurysmal. The etiology of acquired RAVF may be idiopathic or secondary. Idiopathic RAVF may develop when pre-existing renal aneurysms form shunts with adjacent renal segmental veins. Secondary RAVF is caused by iatrogenic injury such as renal biopsy, percutaneous nephrostomy or nephrectomy, penetrating renal trauma such as bullet or stab wounds, or blunt renal injury incurred during an accident or a fall [4-9]. Among these, renal biopsy is the most common cause of secondary RAVF [6]. Moreover, renal malignant tumors may cause formation of shunts between pseudoaneurysms and renal segmental veins, resulting in RAVF [10].

The clinical symptoms of cirsoid-type RAVF include hematuria, bladder tamponade due to coagulated blood in urine, and flank pain. These symptoms result from development of cirsoid-type RAVF from a nidus in the submucosa of the renal pelvis. In contrast, aneurysmal-type RAVF is generally asymptomatic; therefore, it is often incidentally detected by abdominal CT or ultrasonography. Aneurysmal RAVF sometimes presents with symptoms of congestive heart failure if the shunt flow is high. In many cases, aneurysmal RAVF cannot be detected until symptoms of circulatory syndrome become evident. Hematuria is present in 62% cases with cirsoid-type RAVF; however, it is present in only 27% cases with aneurysmal-type RAVF [11]. In the case reported here, acquired RAVF of cirsoid type with renal segmental artery aneurysms was identified. No prophylaxis antibiotic use may lead to sepsis condition. Therefore, prophylaxis antibiotic should have taken into consideration since the day of admission when plain abdominal CT revealed an evidence of hematoma in the pelvis renalis whether it was associated with renal vascular diseases or not.

Renal arteriography is the gold standard for diagnosis of RAVF. Depiction of renal veins in the early stages of the arterial phase confirmed the diagnosis of RAVF in our case. In recent years, advances in multidetector row CT have improved the depiction of renal veins in the early stage of the arterial phase. In addition, visualization of feeding arteries and draining veins using three-dimensional imaging techniques enables diagnosis of RAVF in a minimally invasive way. Magnetic resonance angiography without contrast medium and color Doppler ultrasonography may also be helpful in the diagnosis of RAVF [12,13]. With the improvement of imaging for diagnostic purposes, an increasing number of cases of asymptomatic RAVF may be found.

RAVF with hematuria, flank pain, and symptoms of congestive heart failure requires prompt treatment. Treatment of asymptomatic aneurysmal-type RAVF with high shunt flow is also recommended for the prevention of circulatory complications. Both conservative and surgical treatments such as partial nephrectomy, total nephrectomy, renal autotransplantation, and transarterial embolization may be utilized. In recent years, transarterial embolization has become the gold standard for treatment of RAVF because it is minimally invasive. Embolic materials used for transarterial embolization include gelatin sponge, metallic coil, absolute ethanol, lipiodol, and n-butyl 2-cyano crylate. In the case presented here, metallic coils were chosen for successful treatment of RAVF.

## Conclusion

Here we have reported a case of ruptured renal arteriovenous malformation, which was successfully treated by catheter embolization. In this case, plain abdominal CT suggested hematoma in the pelvis; therefore, renal pelvis carcinoma or ureteral carcinoma was strongly suspected. However, subsequent contrast-enhanced abdominal CT suggested renal arteriovenous malformation with aneurysms. Renal angiography confirmed the diagnosis. When encountering hematuria, contrast-enhanced abdominal CT should be considered for accurate and rapid diagnosis.

## Consent

Written informed consent was obtained from the patient for publication of this Case report and any accompanying images. A copy of the written consent is available for review by the Editor-in-Chief of this journal.

## Competing interests

The authors declare that they have no competing interests.

## Authors’ contributions

Wrote the first draft of the manuscript: NT, YN. Contributed to the writing of the manuscript: NT, YN. Agree with manuscript results and conclusions: NT, YN. Jointly developed the structure and arguments for the paper: NT, YN. Made critical revisions and approved final version: NT, YN. All authors reviewed and approved of the final manuscript.
